# Face-n-Food: Gender Differences in Tuning to Faces

**DOI:** 10.1371/journal.pone.0130363

**Published:** 2015-07-08

**Authors:** Marina A. Pavlova, Klaus Scheffler, Alexander N. Sokolov

**Affiliations:** 1 Department of Biomedical Magnetic Resonance, Medical School, Eberhard Karls University of Tübingen, Tübingen, Germany; 2 High-Field Magnetic Resonance Center, Max Planck Institute for Biological Cybernetics, Tübingen, Germany; 3 Department of Women’s Health, Women’s Health Research Institute, University Hospital, Eberhard Karls University of Tübingen, Tübingen, Germany; Harvard Medical School, UNITED STATES

## Abstract

Faces represent valuable signals for social cognition and non-verbal communication. A wealth of research indicates that women tend to excel in recognition of facial expressions. However, it remains unclear whether females are better tuned to faces. We presented healthy adult females and males with a set of newly created food-plate images resembling faces (slightly bordering on the Giuseppe Arcimboldo style). In a spontaneous recognition task, participants were shown a set of images in a predetermined order from the least to most resembling a face. Females not only more readily recognized the images as a face (they reported resembling a face on images, on which males still did not), but gave on overall more face responses. The findings are discussed in the light of gender differences in deficient face perception. As most neuropsychiatric, neurodevelopmental and psychosomatic disorders characterized by social brain abnormalities are sex specific, the task may serve as a valuable tool for uncovering impairments in visual face processing.

## Introduction

Faces represent valuable signals for social cognition and non-verbal communication. In accordance with widespread beliefs, a wealth of findings shows that females are more proficient in recognition of facial emotions [1–10]. This advantage is already noticeable early in perceptual development [2]. At identifying emotions in photographs (choosing a picture that corresponds to a described emotion) girls aged 3.5 years are as good as 5-year-old boys [11]. In females, activation in cortical and subcortical brain structures is more bilaterally distributed presumably enabling contribution of both hemispheres to facial affect recognition [12–15].

Yet accuracy in facial affect recognition most likely depends on the emotion type. Women are better in recognizing facial expressions of fear and sadness [16,17], whereas males have an edge in identifying anger [16,18,19]. Anger expressed by male actors is more accurately perceived than anger portrayed by female expressers [19,20]. Both behavioral and the amygdala responses to threat-related face expressions (fear and anger) in young men are correlated with testosterone level [21]. Females experience happy faces as more pleasant and sad faces as more unpleasant than do males [22]. Females tend to better recognize emotions from faces than from voices, whereas males exhibit the opposite tendency [23].

Gender differences have not always been observed in the explicit recognition of dynamic (more natural) face expressions [24–26]. In women, dynamic expressions have been associated with higher intensity ratings for anger and happiness whereas in men, influence of dynamics was limited to anger [27]. Furthermore, women exhibit higher sensitivity to facial emotional expressions in dynamic as compared with static displays (photographs) [27]. Autistic individuals are impaired on visual processing of dynamic faces [28], and have increased functional magnetic resonance imaging, fMRI, brain activation in the fusiform gyri in response to emotional faces [29]. Females with and without Asperger syndrome are better at recognizing emotions from dynamics faces than males [23]. This report is in accord with the data indicating that autistic traits are linked to reduced face identity and face recognition in men but not in women [30]. Overall, the findings suggest that facial affect recognition mechanisms and their impairments are gender specific.

It remains unclear whether females excel on non-affective face perception. Boys and girls perform equally well on face processing task—finding a line drawing of a target face among an array of distracters [31]. Yet females outperform on facial detection task (recognition of a face as a face) and facial identity discrimination [32,33]. By contrast, males excel both on the Mooney Face Test [34] and in detecting the Mooney faces, two-tone face depictions, among two similar distracters [35]. The Mooney faces, however, seem to be subject of rather unique perceptual and neural processing [34,36].

Actor gender-based effects may contribute to female superiority in non-affective face processing: Females are better in recognizing faces, especially their own-gender faces [37], and 9-year-old girls outperform boys in recognition of female but not male faces [38]. Yet males are reported to be *wired for her face*: males exhibit attentional bias toward female faces, and female faces elicit stronger brain response in the face selective part of the fusiform gyri [39]. Other findings indicate that both male and female observers are more efficient in recognition of female faces, and several brain areas, including the hippocampal region, exhibit greater fMRI response to female compared to male faces [40].

**Fig 1:**
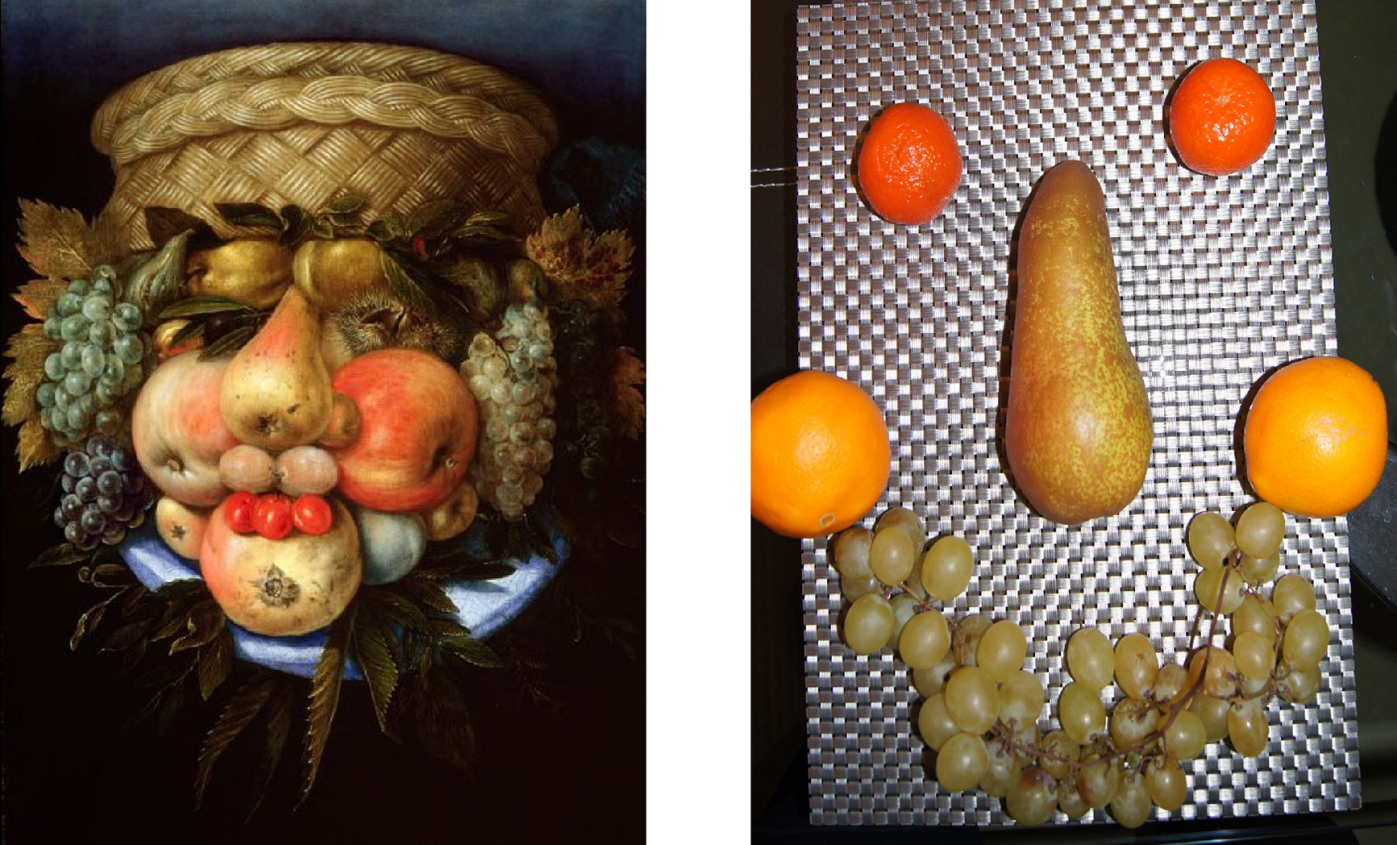
Examples of the Giuseppe Arcimboldo style. Left: “Reversible Head with Basket of Fruit” painting by Giuseppe Arcimboldo (1526–1593), an Italian painter best known for creating fascinating imaginative portraits composed entirely of fruits, vegetables, plants and flowers (image source Artdaily.org; public domain). Right: The portrait of one of the authors of this paper (ANS) created by another author (MAP) in a manner slightly bordering on the style of Giuseppe Arcimboldo.

The present study is aimed at investigation of gender differences in tuning to faces. For this purpose, a new Face-n-Food task has been created. We presented healthy adult females and males with a set of food-plate images composed of food ingredients (fruits, vegetables, sausages, etc.) in a manner slightly bordering on the style of Giuseppe Arcimboldo (1526–1593), an Italian painter best known for creating fascinating imaginative portraits composed entirely of fruits, vegetables, plants and flowers (Fig 1). It appears that typically developing adults and children have an entire bias for seeing faces and their spontaneous and effortless recognition in the Arcimboldo-like images. In other words, healthy individuals are well tuned to faces in such images.

## Methods

### Participants

One hundred four young adults, students of the University of Tübingen, participated in the study. They were assigned to one of two groups: a pilot group and an experimental one. The pilot group consisted of 40 participants (age range 19–35 years, 20 females, 20 males). The experimental group included 64 participants (age range 18–36 years; 34 females aged 22±1.46 years (median± 95% confidence interval), and 30 males, aged 22±0.7 years; with no gender differences in age, Mann-Whitney test, *U* = 460.5, n.s.). Participants were run individually. For the absence of acute hunger (that could potentially intensify observers’ focus on food ingredients), they were tested right after having a snack. All participants had normal or corrected-to-normal vision. None had a history of neurological or psychiatric disorders including autistic spectrum disorders (ASD) or regular medication. None had previous experience with such tasks. The study was conducted in line with the Declaration of Helsinki and was approved by the local Ethics Committee at the University of Tübingen Medical School. Informed written consent was obtained from all participants. Participation was voluntary, and the data were processed anonymously.

### Task and procedure

**Fig 2:**
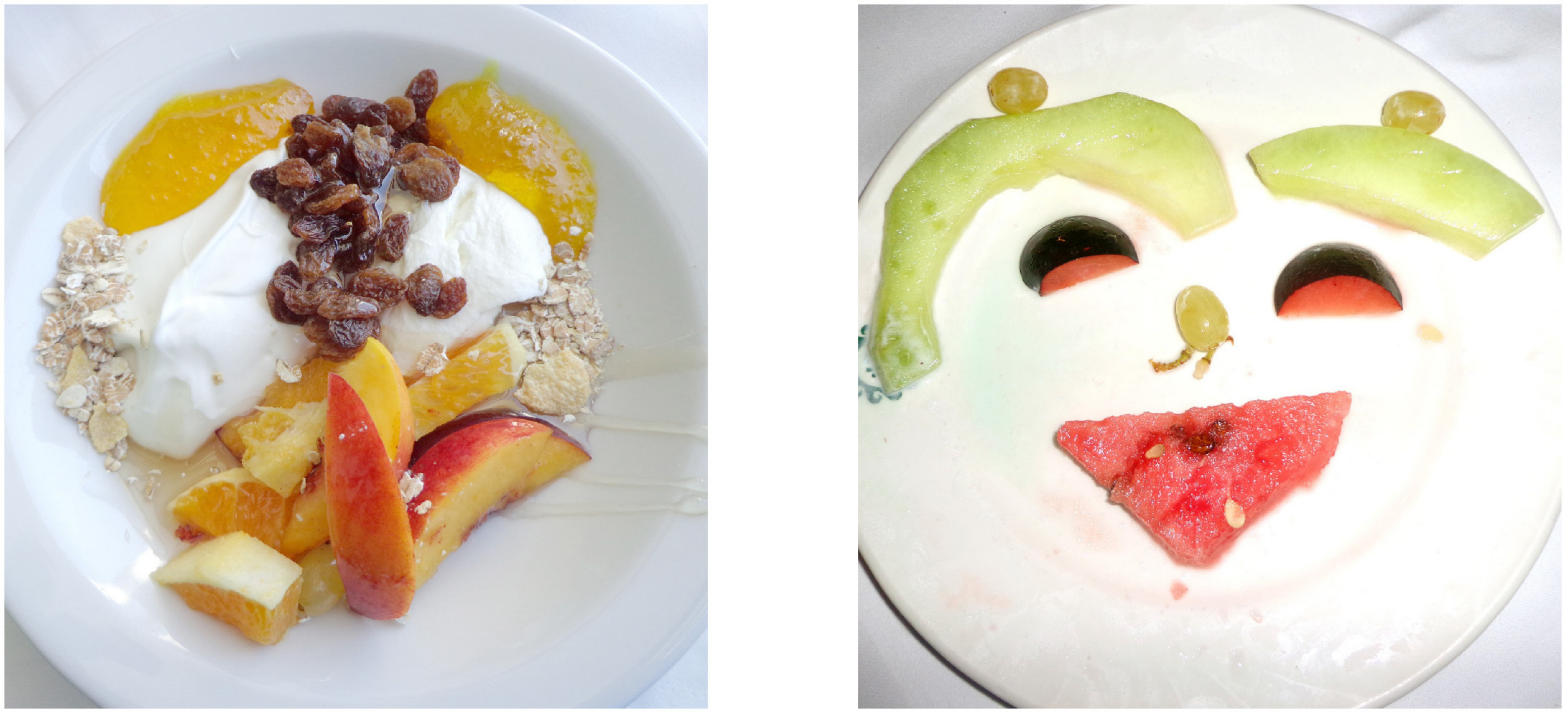
Examples of the Face-n-Food images. The least resembling face (left panel) and most resembling face (right panel) images from the Face-n-Food task.

The Face-n-Food task was administered to participants. For this task, a set of ten food-plate images were created that were composed of food ingredients (fruits, vegetables, sausages, etc.) and resembled faces. The images slightly border on the Giuseppe Arcimboldo style. As a first step, the pilot group of participants had to arrange the set of ten images according to their recognizability as a face from the least (number 1) to most recognizable one (number 10; see Fig 2). Then the experimental group was presented with the set of these images in the predetermined order from the least to most resembling a face (images 1 to 10). On each trial, participants had to perform a spontaneous recognition task: they were asked to briefly describe what they saw. On each trial, no immediate feedback was provided. To avoid time pressure that could potentially cause stress and negative emotional and physiological reactions blocking cognitive processes, there was no time limit on the task. With each participant, the testing procedure lasted for about 15–20 min.

## Results

Participants either described a food-plate image in terms of food composition (typical food responses: Wurst, Joghurt, Obst, eine merkwürdige Mischung—sousages, jogurt, fruits, an odd mixture; Jemand mag keine Wurst im Essen—Somebody does not like sausages on a plate; eine Kalorienbombe—a heart attack on a plate) or as a face (typical face responses: Picasso beim Kochen? Ein komisches Gesicht—Picasso by cooking? An odd face; ein erstauntes oder erschrockenes Gesicht—an astonished or scared face; eine dicke Oma—a thick grandma; ein Gesicht, schreit um Hilfe—a face, appeals for help; Slonge Bob; ein Gesicht, sieht böse aus wegen der Augenbrauen, weint gleich—a face, looks angry because of eyebrows, will cry soon; eine Diva, stark geschminkt—a diva, with strong make-up; das Gesicht von Miss Piggy—Miss Piggy’s face). Other than face or food responses (e.g., sieht wie ein Kleid aus—looks like a dress) were given extremely rarely with a rate of 0.016.

**Fig 3:**
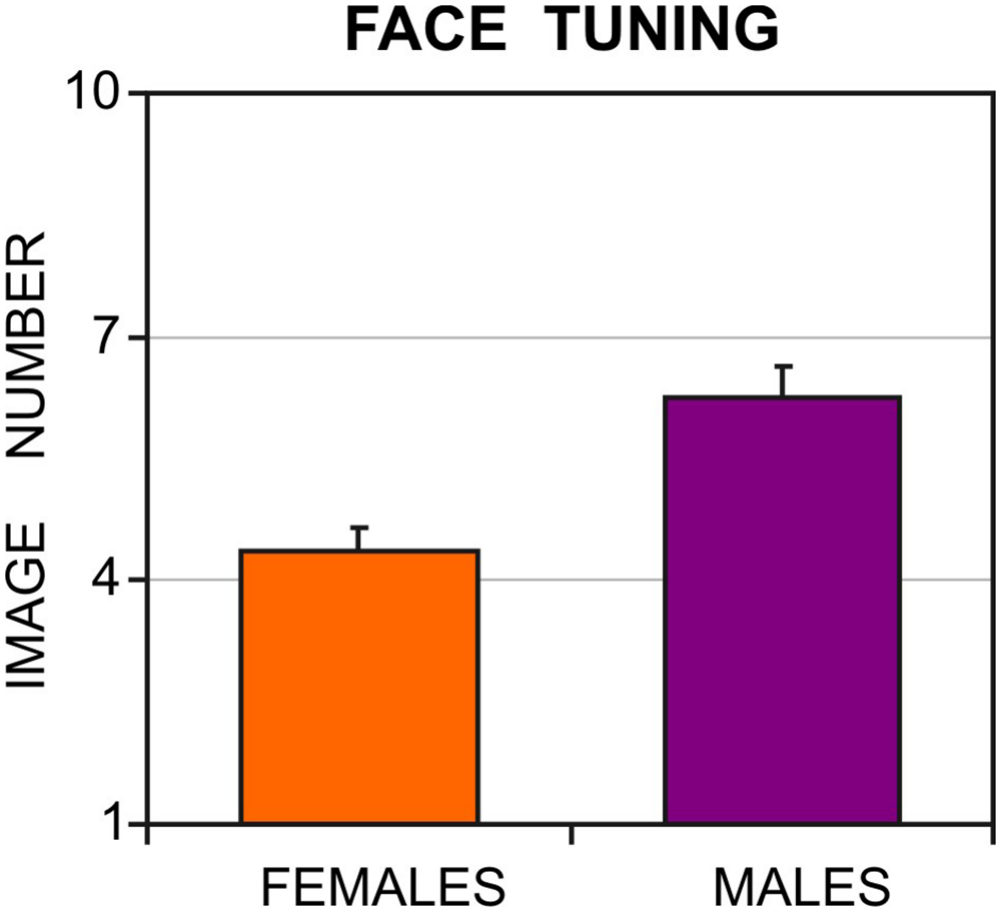
Gender difference in tuning to faces. In the Face-n-Food task, females more readily recognize images as a face than males. Vertical bars represent SEM.

Fig 3 shows the average image number, on which resembling a face on the Face-n-Food task (face response) was initially reported, separately for female and male participants. As can be seen from this figure, females more readily recognized the images as a face than males: females reported seeing a face on average on 4.35±1.69 (mean±standard deviation, SD) image, whereas males gave the first face response on average only on 6.27±2.03 image. The gender difference is highly significant (t(62) = 3.83, *p* < 0.0002, two-tailed, with an effect size Cohen’s *d* = 1.025). This gender difference cannot be explained by a stronger experimenter expectancy of females (when participants try to interpret the purpose of the experiment and to please the experimenter) or by entire bias towards seeing faces, because 14 out of 34 females gave at least one non-face response on subsequent images after their initial face response. By contrast, 27 out of 30 male participants reported seeing a face on all subsequent images. As not all participants reported seeing a face on all subsequent images, we performed an additional analysis on the total number of images recognized as a face by females and males. The outcome indicates that percentage correct is 60.09±14.85% (mean±standard deviation) for females and 46±20.94% for males. The gender difference in the percentage of face responses is significant (t(62) = 2.97, *p* < 0.002, two-tailed, with Cohen’s *d* = 0.82). Although this analysis provides an additional support for female superiority on the Face-n-Food task, an initial face report appears to be a more proper measure of tuning to faces. Subsequent reports may be potentially affected by other factors such as low or high self-confidence or a kind of aha!-effect.

**Fig 4:**
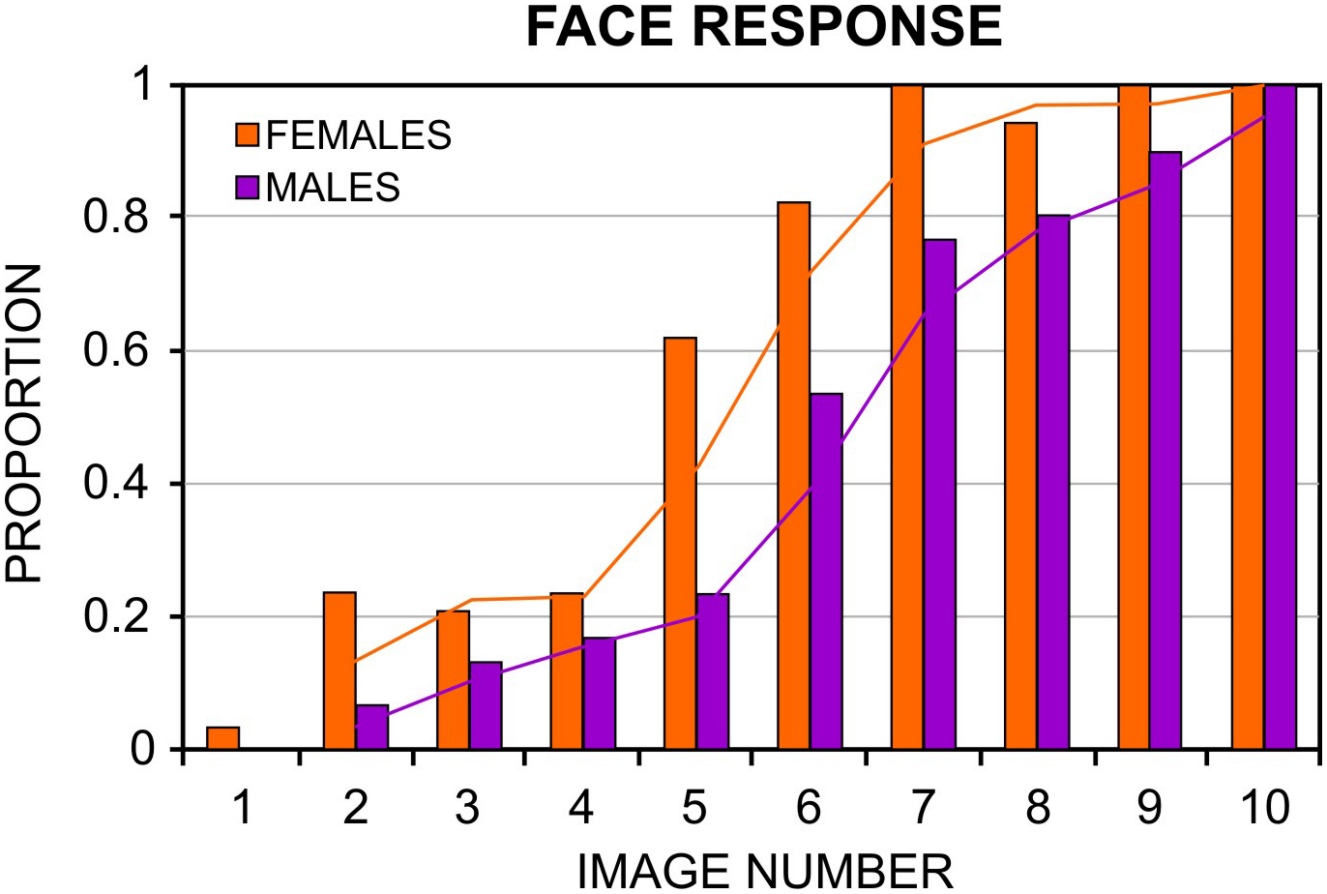
Proportion of face responses to each image in the Face-n-Food task for female and male participants. The image number reflects its recognizability as a face (1—the least recognizable, 10—the most recognizable). Fitted trend curves represent moving average of face response proportion across the images. Females not only earlier report seeing a face and give on overall more face responses, but also faster reach a ceiling level of performance.

Fig 4 represents the proportion of face responses for each Face-n-Food image for females and males. As seen from this figure, females not only earlier report seeing a face, give more face responses, but also faster than males reach a ceiling level of performance giving the maximal number of face responses. This is confirmed by statistical analysis performed on individual frequencies of face responses as dependent measure and images used. The analysis reveals a highly significant effect of gender (χ2 (1; 64) = 52.45, *p* < 0.0001). The gender by image interaction (χ2 (1; 64) = 14.01, *p* < 0.0002) is also highly significant indicating that the slope of the fitted curves for females is much steeper than for males.

## Discussion

Although mounting evidence points to females’ advantage in facial affect recognition, it is unclear whether females are also better tuned to faces. By using the newly created Face-n-Food task consisting of a set of food-plate images comprising food ingredients (fruits, vegetables, sausages, etc.) in a manner slightly bordering on the Giuseppe Arcimboldo style, we investigated gender differences in tuning to faces. The findings indicate females’ superiority in recognizing food-plate images as a face: they report resembling faces on images that are still described by males in terms of food ingredients only. Females not only earlier report seeing a face, but also give on overall more face responses and faster achieve the ceiling level of performance. The outcome reveals that females are likely to have an advantage not only in emotional face processing: they are also better tuned to faces, at least, on the Face-n-Food task.

The present data dovetail with reports on superior skills of females on some other essential components of visual social cognition such as body language reading (the ability for understanding emotions, intentions, drives, and dispositions of others through body motion). Females are faster in discrimination of emotional from neutral body motion [41]. Both facial affect processing and body language reading [42,43] appear to be profoundly modulated by the type of portrayed emotion. Brain fMRI activity elicited by threatening facial and bodily expressions is modulated by observer’s gender [25]. Females are also more accurate in recognition of point-light neutral body motion (such as walking or jumping on the spot) [41]. Neuroimaging reveals gender dependent modes in the brain response to neutral body motion even in the absence of behavioral gender differences. In females, early magnetoencephalographic, MEG, activation occurs over the right parietal, left temporal, and right temporal cortex, a core of the social brain, whereas in males, the boosts of later activation are greater over the right frontal and occipital cortices [44]. In females, increased fMRI activity is found during viewing of point-light body motion (waving, playing pat-a-cake and peek-a-boo) over the regions constituting the social brain (the temporal pole, medial temporal gyrus, cerebellum, and amygdala) [45]. Overall, observers demonstrate greater ease in judging the neutral actions compared to judging emotional body language. When healthy adults judge emotions represented by stick human body postures, patterns of fMRI activity are sex specific, in particular, over the left anterior insula, left dorsal premotor cortex and right superior parietal lobule [46]. Yet sex differences are not evident in the neural circuitry underpinning visual processing of social interaction in Heider-and-Simmel movies, but rather in the regions engaged in perceptual decision making: In males, the MEG oscillatory induced gamma response boosts later than in females over the left prefrontal cortex [47]. It appears that females anticipate social interaction predicting others’ actions ahead of their occurrence, whereas males require accumulation of more sensory evidence for reaching proper decisions. Future research should be directed at uncovering sex differences in the social brain. Among other issues to clarify is whether neural circuits underlying tuning to faces are sex specific.

The present findings on female superiority in tuning to faces are in agreement with previous reports: females outperform on facial detection task (recognition of a face as a face) and facial identity discrimination [32,33]. At first glance, our data contradict male benefit in visual processing of the Mooney faces [34,35]. These images, however, seem to be subject of rather unique perceptual and neural processing [34,36]. Moreover, to find any facial feature such as an eye or nose, one must first holistically perceive the Mooney image as a face [48]. One can speculate that right-hemisphere dominant fusiform brain activation reported in men favors holistic face processing [49]. Yet identification of the precise nature of gender effects in tuning to faces is beyond the scope if this study. Methodological issues (such as the nature of stimuli: static or dynamic displays; real or arty faces; different stages of face processing addressed; and various task demands that may be non-specific to face encoding itself) may be of potential importance for the outcome of studies aimed at uncovering gender effects in face processing.

Although the Face-n-Food images had been created without any purpose to appear gender specific, they could potentially produce a gender related impression. The cues that may affect encoding face gender-specificity (such as a longer nose and oval face shape in masculine face, thinner eyebrows, bigger eyes and lips in feminine faces; or presence of a smiling expression: across cultures, women smile more often [50]) are minimized or totally absent in the food-plate images. However, clarification of the issue of whether the Face-n-Food images elicit gender related impression requires experimental proof.

The present work may be considered as a first step towards putting the Face-n-Food task into clinical setting. Most neuropsychiatric, neurodevelopmental and psychosomatic disorders are characterized by impairments in visual social cognition, non-verbal communication, body language reading, social competence and social interaction with others [51]. Face perception and facial emotional assessment of a social counterpart is of particular importance for adaptive social behavior. Most diseases related to impairments in visual social cognition are gender-specific: females and males are differently affected in terms of clinical picture, prevalence, and severity. Females are more often affected by anxiety disorders with a ratio of 2:1 or even 3:1, and gender differences increase with age [52,53]. Depression is approximately twice as common in females as in males [54]. By contrast, males have a higher risk for developing autistic disorders, with a ratio of about 4:1 [55] or even higher. Neuroanatomy of autism is sex specific [56]. Schizophrenia occurs 1.4 times more frequently in males than females, and the onset of disease is earlier in men [57]. Males are at a 14–20% higher risk for premature birth and of its complications in the brain development and cognition [58]. Males are also more often affected by attention deficit hyperactivity disorders, ADHD [59].

Visual social cognition, body language reading and different aspects of face processing is altered in most of these disorders [30,60,61]. Face perception is reported to be impaired in individuals with autistic traits with different gender impact: face identity and face recognition are stronger impaired in autistic males [29,30]. In autistic girls, but not boys, atypical face-sensitive component of the event related potentials (ERP), N170, is associated with symptom severity [62]. Preterm born children are also reported to experience difficulties in face processing [63,64], but gender impact on different aspects of face perception and other components of visual social cognition is largely unknown. As indicated by fMRI, during facial emotional categorization young females with major depressive disorder, MDD, exhibit hyperactivation, whereas young males display hypoactivation in the precuneous brain area [65]. Several aspects of face processing are also deficient in social anxiety [66]. Women, but not men, with social anxiety are hypersensitive to threat (avoidance-related) and approval-related facial emotional expressions such as fear, sadness and happiness [67]. Face processing and other aspects of social cognition are compromised in ADHD [68]. Boys with ADHD show impairments in response inhibition toward facial expression of anger that is accompanied by reduced P300 amplitude of the ERP [69], and selective difficulties in matching facial expressions to situations [70]. Although facial affect recognition is poor in both schizophrenia and ADHD, brain imaging indicates reduced activity in the medial prefrontal and limbic (amygdala) brain regions in schizophrenia, but more localized loss of activity in these regions in ADHD [71].

Last but not least, there are pronounced gender differences in eating disorders with a higher risk for developing eating abnormalities in women. Lifetime prevalence is about 0.9% in females to 0.3% in males in anorexia nervosa, and 0.5% in females to 0.15% in males in bulimia nervosa [72]. Categorization of facial emotions, in particular, negative emotions (fear and anger), but also other facial expressions (happiness and surprise) are reported to be impaired in females both in anorexia nervosa and bulimia nervosa [73–78] with a tendency to mislabel anger for sadness [78]. In eating disorders, alterations in brain processing of emotional faces are also reported [76,77]. For example, in females with bulimia nervosa, face-specific N170 amplitude of the ERP is lower for angry faces [77]. Yet it is unclear whether individuals with eating disorders experience difficulties in tuning to faces, in facial affect recognition only, or in both aspects of face processing. As the Food-n-Face task can potentially differentiate observers’ focus either on faces or food, in future work, we intend to prove the viability of this task as a quick and reliable indicator of tendencies to exhibit eating abnormalities.

A tendency of seeing faces in ambiguous images such as clouds or ink blots that contain elements resembling those of a face, is sometimes called face pareidolia. The time-course of the brain MEG response in the right ventral face fusiform area (FFA) during processing of faces and face-like images is rather similar: the brain is likely to be hardwired to detect the presence of a face as quickly as possible, rather than to process face-like images later on under influence of top-down mechanisms [79]. Yet the right superior temporal sulcus differentiates between faces and face-like images. Functional MRI also indicates that the right FFA is active during perception of noise images containing components resembling a face: images even with the slightest face cues are interpreted as faces [80].

Further step in elaborating the Face-n-Food images in relation to sex differences in the social brain and cognition would be recording the brain activity, in particular, by means of fMRI, in different patient population groups. Specific topographic patterns of activity within the neural circuitry underpinning facial processing including the FFA, can add essential information about processing of these images in the typical and atypical social brain.

## Conclusions

The outcome of the present work indicates that females are more sensitive to faces represented by a composition of food ingredients in a manner bordering on the Guiseppe Archimboldo style. Females more readily recognize the images as a face: they not only earlier report resembling a face on images that are still described in terms of food by males, but also give on overall more face responses, and faster reach the ceiling level of face recognition. As most neuropsychiatric, neurodevelopmental and psychosomatic disorders characterized by social brain abnormalities are gender specific, the task may serve as a valuable tool for uncovering impairments in visual face processing.
